# Carbonate formation and fluctuating habitability on Mars

**DOI:** 10.1038/s41586-025-09161-1

**Published:** 2025-07-02

**Authors:** Edwin S. Kite, Benjamin M. Tutolo, Madison L. Turner, Heather B. Franz, David G. Burtt, Thomas F. Bristow, Woodward W. Fischer, Ralph E. Milliken, Abigail A. Fraeman, Daniel Y. Zhou

**Affiliations:** 1 University of Chicago, Chicago, IL USA; 2 University of Calgary, Calgary, Alberta Canada; 3 NASA Goddard Space Flight Center, Greenbelt, MD USA; 4 NASA Ames Research Center, Moffett Field, CA USA; 5 California Institute of Technology, Pasadena, CA USA; 6 Brown University, Providence, RI USA; 7 Jet Propulsion Laboratory, California Institute of Technology, Pasadena, CA USA

**Keywords:** Planetary science, Planetary science

## Abstract

The cause of Mars’s loss of surface habitability is unclear, with isotopic data suggesting a ‘missing sink’ of carbonate^1^. Past climates with surface and shallow-subsurface liquid water are recorded by Mars’s sedimentary rocks, including strata in the approximately 4-km-thick record at Gale Crater^2^. Those waters were intermittent, spatially patchy and discontinuous, and continued remarkably late in Mars’s history^3^—attributes that can be understood if, as on Earth, sedimentary-rock formation sequestered carbon dioxide as abundant carbonate (recently confirmed in situ at Gale^4^). Here we show that a negative feedback among solar luminosity, liquid water and carbonate formation can explain the existence of intermittent Martian oases. In our model, increasing solar luminosity promoted the stability of liquid water, which in turn formed carbonate, reduced the partial pressure of atmospheric carbon dioxide and limited liquid water^5^. Chaotic orbital forcing modulated wet–dry cycles. The negative feedback restricted liquid water to oases and Mars self-regulated as a desert planet. We model snowmelt as the water source, but the feedback can also work with groundwater as the water source. Model output suggests that Gale faithfully records the expected primary episodes of liquid water stability in the surface and near-surface environment. Eventually, atmospheric thickness approaches water’s triple point, curtailing the sustained stability of liquid water and thus habitability in the surface environment. We assume that the carbonate content found at Gale is representative, and as a result we present a testable idea rather than definitive evidence.

## Abstract (web summary)

A modelling study suggests that Mars had a desert-like climate with intermittent liquid-water oases regulated by a negative feedback among solar luminosity, liquid water and carbonate formation.

## Main

Earth has kept a habitable climate for >3.5 billion years (Gyr). By contrast, Mars has lost surface habitability over time, a contrast that demands explanation. The main climate-regulating greenhouse gas for modern Earth, Venus and Mars is carbon dioxide (CO_2_). The abundance of (inorganic) carbon in Earth’s atmosphere and ocean is the small difference between time-integrated outgassing from volcanoes, versus time-integrated carbon sequestration, mainly as carbonate in sedimentary rocks^6^. The necessary close balance between (mainly carbonate) sedimentary-rock sinks and carbon sources suggests that a stabilizing feedback has regulated carbonate formation, and thus atmospheric CO_2_ content and climate, over billions of years on Earth^7^. This concept underpins the circumstellar habitable zone hypothesis^8^.

For Mars, it was thought that a thicker CO_2_ atmosphere corresponding to the early habitable era would be entombed as carbonate in sedimentary rock^9,10^. However, neither global-scale orbital spectroscopy^11–13^ nor site-specific initial rover exploration^14–16^ found much carbonate. Nevertheless, isotopic analysis suggests a missing near-surface carbonate sink, because post-3.5-billion-years-ago (Ga) loss of a thicker atmosphere primarily via escape to space would make the atmosphere richer in ^13^C than is observed^1,17^.

Recently the situation has changed, as both of NASA’s active Mars rovers, Curiosity and Perseverance, have made major carbonate finds^4,18^.

Among sedimentary-rock depocentres, one of the thickest (approximately 4 km) and most diverse records is the Mars Science Laboratory (MSL) Curiosity rover’s field site, Aeolis Mons (‘Mount Sharp’) in Gale Crater^2^. Ascending Mount Sharp, MSL has thus far identified predominantly aeolian deposits within the magnesium-sulfate-bearing unit, cemented and altered by diagenetic fluids^19^. This predominantly aeolian interpretation is consistent with inferences from orbit and from other landing sites^20,21^. Overall, the data indicate that Mars did not have a billion-year-long climate that supported rivers and lakes continuously, because Mars is not as chemically weathered or physically eroded as would be expected had rivers and lakes persisted globally for that long^3,22^.

Recently, as MSL climbed through the layer-cake stratigraphy of Mount Sharp, it began to find rocks with high (5–11 wt%) concentrations of ‘cryptic carbonates’, deposits that were not initially found from orbit^4^. Orbital detection of carbonates relies on spectral absorptions at 2.3 μm and 2.5 μm (ref. ^11^); however, carbonate spectral features can be obscured by dust or confused with features produced by other minerals^11,12^. These high concentrations were first found at a topographic level of −3,853 m (about 11% of the way to Mount Sharp’s summit) and span 5 drill samples so far covering about 180 m of topographic elevation. The topmost drill sample is from a block transported by a flow from higher elevation, so the true topographic span of the carbonate-rich rocks is almost certainly more than 200 m. Geologic context and modelling suggest that a likely source of the carbon was atmospheric CO_2_ (ref. ^4^). Reference ^4^ multiplies the observed carbonate concentration by the area of laterally continuous sulfate facies and rhythmite facies sedimentary rocks on Mars (>2.7 × 10^6^ km^2^) and an inferred rock density of 2,300 ± 130 kg m^−3^ (ref. ^23^) to suggest a carbonate content of 6.2 × 10^14^ kg per metre of stratigraphy. Multiplying by the molar mass ratio of CO_2_ to siderite (FeCO_3_), which is 44:116, and correcting for Mars gravity, the CO_2_ sequestered is 0.03–0.06 mbar per metre.

Meanwhile, at Jezero’s rim, carbonate-rich sedimentary rocks have now been confirmed in situ, and apparently formed on the shores of an ancient lake^18^. These materials probably formed post-3.5 Ga, and extend for tens of kilometres and around the rim^24^.

Thus, on Mars as on Earth, carbonate formation and sedimentary-rock formation (and early-diagenetic alteration) can be coupled. This coupling motivates re-evaluating the hypothesis that Mars’s sedimentary rocks were not just a witness, but also a driver, of surface conditions becoming less habitable.

**Extended Data Fig. 1:**
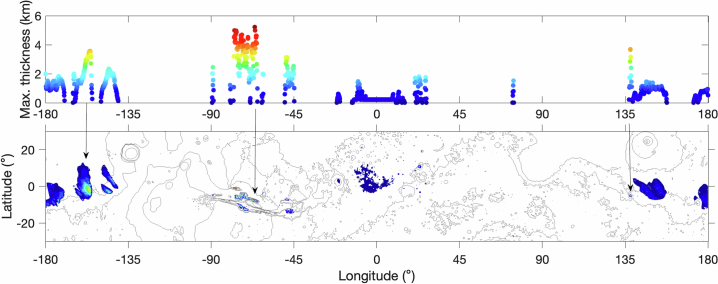
Distribution of sedimentary rock mounds on Mars. Top: maximum thickness of mapped sedimentary rock as a function of longitude. Arrows (from right to left): Mount Sharp in Gale Crater; thick mounds in Valles Marineris; and Eumenides Dorsum. Bottom: map of sedimentary rock mounds. Total area = 2.4 × 10^6 ^km^2^, total volume = 1.4 × 10^6^ km^3^. For 10 wt% FeCO_3,_ this volume corresponds to 30 mbar pCO_2_ drawn down. Mapping procedure is similar to that in ref. ^4^.

Surface and near-surface liquid water is needed for a habitable climate; other essential ingredients for habitability (for example, organic matter) were present on Mars^25,26^. Evidence of a habitable climate on early Mars is recorded by sedimentary rocks, which give space and time constraints on surface conditions^21^. We focus on post-3.5-Ga Mars, when Mount Sharp’s magnesium-sulfate-bearing unit and overlying light-toned unit (termed ‘rhythmite’, so-called because of its regular layer thickness) were formed. Together, these rock types (‘orbital facies’) cover 2 × 10^6^ km^2^ (Extended Data Fig. 1)—likely a lower bound on the spatial extent of liquid water, as their lithification probably involved liquid water. However, local areas of water availability, here referred to as oases, were patchy, as much of Mars was dry after about 3.5 Ga (for example, ref. ^27^). Indeed, almost all post-3.5-Ga sedimentary-rock outcrops (by volume) are in 3 longitudinal pockets <10° from the equator (Methods). These rocks formed over a span of more than 1 Gyr, and apparently as late as 0.5 Ga (ref. ^28^). Sedimentation rates estimated from rhythmic strata are 0.03–1.5 m kyr^−1^ (refs. ^29,30^). Large unconformities record basin-scale (global?) pauses in sedimentation (for example, ref. ^2^).

**Extended Data Fig. 2:**
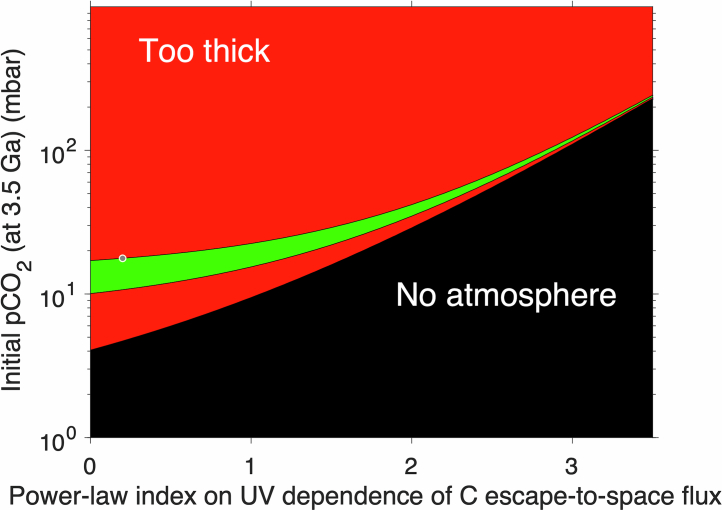
Fine-tuning of initial pCO_2_ and/or escape rate dependence (power law exponent on XUV) is needed to allow escape-to-space to match the observation of a thin atmosphere on Mars (green zone). Most choices give either the wrong atmospheric thickness (red zones) or no atmosphere at all (black zone). By contrast, carbonate formation naturally adjusts the pCO_2_ to about the modern observed value (as previously suggested^5^). Isotopic data also suggest escape-to-space is not a dominant post-3.5 Ga atmospheric loss channel and that there is a “missing sink” of carbonate^1,17^.

Models of post-3.5-Ga Mars climate evolution incorporating only atmospheric loss to space can explain the decline in surface water^27^ but face a fine-tuning problem^5,31^ (Extended Data Fig. 2) in explaining both the modern CO_2_ inventory^32^ and the extended duration of intermittent surface water^28^. In contrast, models that include carbonate formation (either alone or combined with atmospheric loss) successfully explain all three observations (Extended Data Fig. 2).

Here we use a spatially resolved long-term climate evolution model to study Mars (Methods and Extended Data Table 1). Our model includes chaotic orbital forcing^33^, geographic variations, carbonate formation, escape to space, and both day–night and seasonal cycles in surface temperature, simplifying the surface ice distribution using a parameter, and neglecting horizontal heat transport by the atmosphere (Methods). Runs are initialized at 3.5 Ga and integrated forwards in time from an initial atmospheric pressure of (typically) 60 mbar. The climate state at each time step and each location on the surface determines whether or not liquid water is available; if it is, some carbonates form in proportion to the frequency of warm seasons in which liquid water is available and the atmospheric pressure is reduced. We include a parameterization of escape to space based on the results from refs. ^34,35^ (Extended Data Table 2). Previous work on atmosphere evolution^1,31^ is globally averaged and lacks self-consistent chaotic orbital forcing. As carbonate sequestration in sedimentary rocks requires liquid water, this model centres on water supply (Methods). On post-3.5-Ga Mars, we do not know whether the water source was deep-groundwater upwelling or surface and/or shallow-subsurface water from snowmelt. If groundwater (and not snowmelt) had dominated, the fluctuations would still be orbitally paced, perhaps via evaporation-rate change rather than via temperature change. We focus on snowmelt because a detailed model is available^36^, and the resulting feedback we propose could work for any water source that increases for warmer climates (for example, groundwater upwelling^37^ through taliks that open in warm climates).

## Time evolution

**Fig. 1:**
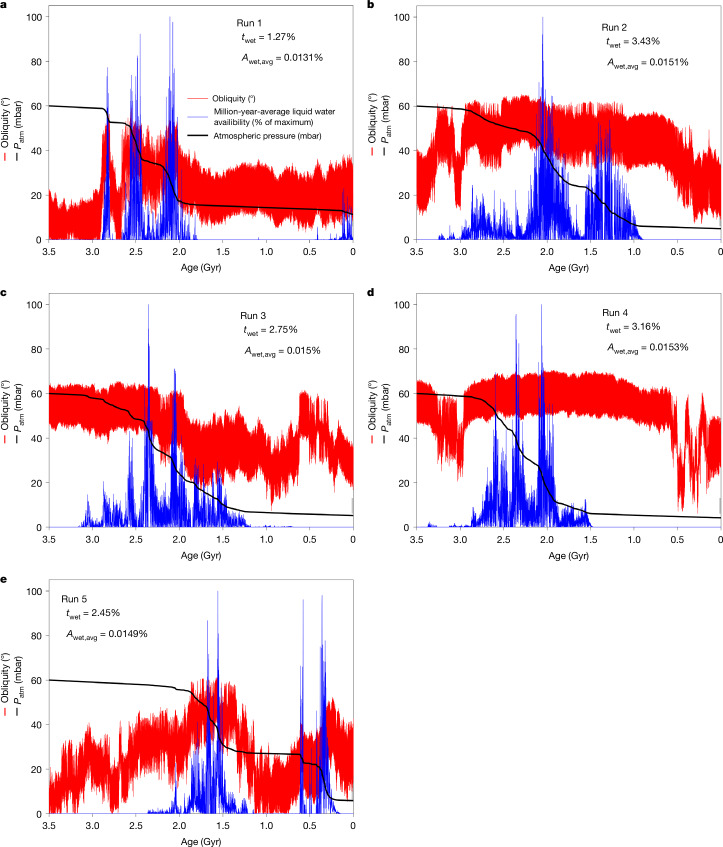
Examples of climate evolution modelled with varying orbital forcing. **a**–**e**, Red line, obliquity; black line, ${p}_{{{\rm{CO}}}_{2}}$; blue line, (1-Myr average) percentage of the maximum area of (seasonal) surface liquid-water availability (which is about 5% of the planet area). *t*_wet_, percentage of time with any liquid water; *A*_wet,avg_, mean surface liquid water cover. The orbital forcing varies between panels, corresponding to uncertainty in Mars’s true past orbital forcing due to Solar System chaos. We infer that Mars’s geology records an imprint of Solar System chaos on planetary climate.

**Fig. 2:**
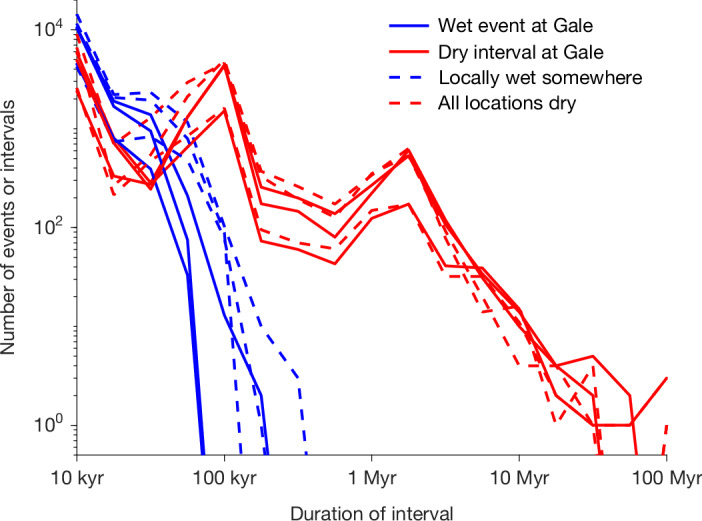
Carbonate formation buffers Mars to a fluctuating habitability state, with orbitally paced wet events and long dry intervals. Blue, histograms of the durations of wet events at Gale and globally; red: durations of dry intervals within the time span of wet events. The three different lines of each type correspond to three different random orbital histories, specifically runs 1–3 from Fig. 1. Globally dry periods are sometimes very long and might drive surface life (had it existed) extinct.

Runs of the model with carbonate formation show a surface liquid-water time span of >1 Gyr, and end near 6 mbar (Fig. 1), with orbitally paced episodes of surface liquid water. Because the episodes are patchy and brief (≲10^5^ yr; Fig. 2), carbonate formation averages <10^−4^ Earth’s rate. Episodes are brief because liquid water requires near-optimal orbital conditions. These outputs are robust to changing initial atmospheric pressure (24–100 mbar) and hold for most simulated orbital tracks (Extended Data Fig. 4). We find that carbonate formation, modulated by brightening sunlight and chaotic orbital forcing, maintains spatially patchy and intermittent (fluctuating) oases of surface liquid water. Specifically, solar brightening allows liquid water, but this permits carbonate formation, which consumes CO_2_, disfavouring liquid water. However, if Mars is too cold for carbonates to form, then solar brightening can warm Mars until melting resumes. This feedback^5^ adjusts the climate so that liquid water is spatially restricted to oases. Moreover, combined with chaotic orbital forcing, very wet conditions do not last long but planet-wide dry conditions do not last indefinitely either (Fig. 2)—that is, fluctuating surface liquid water.

Although volcanic outgassing presumably contributed to the formation of Mars’s atmosphere (4.5 Ga?), we infer that additional volcanic outgassing^38^ of CO_2_ post-3.5 Ga was less significant than carbonate formation (Methods).

## Spatial patterns

**Fig. 3:**
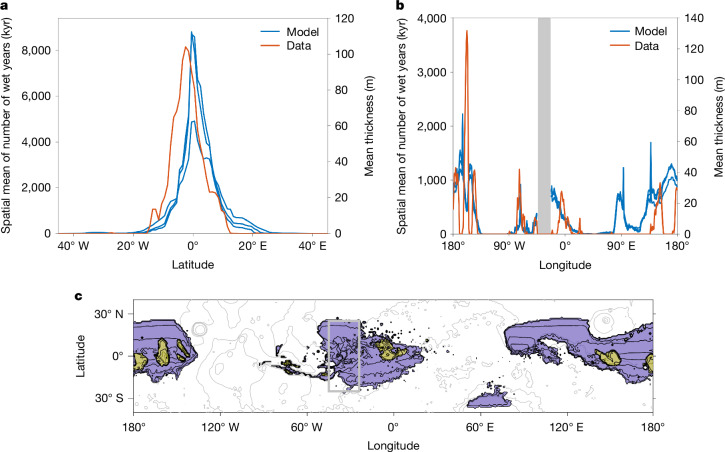
Spatial patterns. **a**,**b**, Latitude (**a**) and longitude (**b**) distribution of sedimentary rock (red) and model-predicted liquid-water availability (blue). Three different model runs, corresponding to runs 1–3 from Fig. 1, are shown. The grey zone in **b** (also shown in **c**) is masked out due to Middle Amazonian catastrophic outburst erosion. It is noted that the model prediction for longitude is 100% due to topography. **c**, Preserved (yellow) and predicted (purple) sedimentary-rock distribution. Run 1, modelled sedimentary rock volume *V*_sed_ (million km^3^), 2.49; CO_2_ sink (mbar), 53.8; maximum modelled sedimentary rock column height *H*_max_ (km), 6.39. The thin grey open contours are topographic contours (2.5-km intervals).

The model predicts liquid water near the equator (Fig. 3) only at high obliquity, and prefers low elevations where greater atmospheric pressure increases greenhouse warming and reduces evaporative cooling. Some liquid water is almost certainly required to form and/or indurate sedimentary rocks, so post-3.5-Ga sedimentary rocks occur only where Mars was relatively wet post-3.5 Ga. Setting aside for now the still-unsolved problem of why few pre-3.5-Ga carbonates are observed^14^, the snowmelt model predicts concentration of post-3.5-Ga sedimentary-rock volume at equatorial latitudes and low elevation, consistent with observations (Fig. 3). Although published groundwater-upwelling models^37^ predict a broader distribution, focusing of late-stage groundwater upwelling at low latitudes by an impermeable cryosphere elsewhere might reconcile groundwater upwelling with the observed distribution of sedimentary-rock volume^39^. Therefore, our carbonate-feedback climate evolution scenario can be reconciled with data using either a top-down or a bottom-up water source.

Erosion during dry periods (including the modern era) means that only the thickest sedimentary-rock deposits persist today. Thus, the volume and area of carbonate-rich sediment may exceed the volume of sedimentary rock retained in the modern rock record.

As an equatorial, low-elevation warm spot, Gale is among the global maxima in predicted wetness (Extended Data Fig. 5). Each main phase of modelled low-latitude wetness occurs at Gale (Extended Data Fig. 6). Thus, Gale is an anchor point for studying Mars carbonate formation and climate change^2^. Today, Gale is among the global maxima in sedimentary-rock thickness (Extended Data Figs. 1 and  5).

## Discussion

The model has limitations. For example, modern topography is used, including sedimentary mounds that exist in impact craters and other topographic basins. This limitation causes anticorrelation between liquid-water predictions and mound topography at small spatial scales (for example, near −160° E; Fig. 3b), as mound tops are less favoured for liquid water than neighbouring topographic moats. The model predicts abundant liquid water near 94° E, which lacks thick sedimentary mounds. Images show that this area underwent lake overspills, implying large amounts of liquid water, which could have eroded sediments. The model does not include shifts in clastic availability (for example, changing volcanic-ash sources), which likely occurred. Finally, deep-sourced groundwater flow (that is, not just a seasonally active layer) has modified sedimentary rocks^16,37^, but this water source is not included in the model. As high (>273 K) annual-mean temperatures are needed for climate to initiate deep-groundwater upwelling, this suggests additional non-CO_2_ greenhouse warming, such as from water-ice clouds^40^. We mask out the area of chaos terrains and catastrophic outburst channels near Valles Marineris, where abundant liquid water is predicted, but sedimentary-rock mounds are not observed. Possible explanations include that liquid-water availability caused the catastrophic outburst erosion (for example, by talik through-connection to overpressured aquifer), precluding or eroding^41^ sedimentary rock.

The cation-limited assumption can be justified as follows. Suppose a seasonal snow supply of 2 cm yr^−1^ meltwater-equivalent, which is plausible^42^, and 25 mbar partial pressure of CO_2_ (${p}_{{{\rm{CO}}}_{2}}$). Then the solubility of CO_2_ is about 3 g kg^−1^, giving 6 g yr^−1^ m^−2^ of CO_2_, not including HCO_3_^−^/CO_3_^2−^. This crude calculation allows up to about 16 g m^−2^ yr^−1^ of FeCO_3_. In 10 Myr, this is 160 tons m^−2^ of FeCO_3_, or a drawdown of 2 bar. This analysis shows that cations and formation kinetics, and not CO_2_ solubility or thermodynamic stability, limit carbonate formation at the relevant sedimentation rates^43–46^.

As well as sedimentary rocks, post-3.5-Ga Mars occasionally had big lakes (for example, ref. ^47^). Lake water could be sourced from favourably oriented slopes^48^, or alternatively an anomalous climate, such as ice-sheet melt-back^49^. The model neglects run-off. This is acceptable as run-off post-3.5-Ga was mostly confined within small basins^50^.

If sediment is indurated (added to the rock column) in wet years, but sediment bypass occurs during dry times, and aeolian input is steady and/or uniform, then the sedimentary-rock thickness is proportional to the number of wet years. This is probably an oversimplification. Nevertheless, the maxima of modelled wetness correspond to thick sedimentary-rock accumulations (Extended Data Fig. 5).

Long globally dry periods (Fig. 2) could extinguish surface life. Thus, in our model framework, the oases are considered uninhabited. However, perhaps increasing temperatures unfroze the cryosphere beneath taliks, allowing inoculation of oases from deep-subsurface aquifers^51^. Eventually, the approach to 6 mbar suppresses both habitability and sedimentary-rock formation.

In contrast to cryptic carbonate not initially seen from orbit (but confirmed by rover) at Gale, some carbonate deposits are known from orbit^13^; this study does not change our understanding of those, but underlines their scientific value.

The cryptic carbonate abundance reported by MSL, multiplied by the global sedimentary-rock volume, is about what is needed (in a simple climate model) to dry out Mars (for example, run 1 corresponds to a CO_2_ drawdown of 54 mbar; Fig. 3b). We predict that this is not a coincidence, because in our model the carbonate drawdown adjusts to dry out Mars (Extended Data Fig. 4). Future exploration with MSL can test this hypothesis. So far, MSL has only found abundant carbonate within five drillholes corresponding to approximately 0.2 km of Mount Sharp’s height. If MSL finds that the Mount-Sharp-averaged carbonate abundance is low (<2 wt%), the model is falsified. This is because (if Mount Sharp is representative of post-3.5-Ga sedimentary rocks on Mars) adding the corresponding CO_2_ back into the atmosphere would not produce liquid water on early Mars. The model outputs many pauses in deposition (Fig. 2), thus predicting many hiatuses and scour surfaces. There should be long time gaps at unconformities—in most model runs, the longest syndepositional unconformity lasts ≥10^8^ years (for example, Fig. 2 and Extended Data Fig. 8). MSL is currently driving towards a major unconformity^2^. Carbonates should persist at Mount Sharp’s top, in the light-toned rhythmite unit, even as groundwater-associated minerals diminish at high topographic elevation. However, continued evidence for surface and shallow-subsurface water is predicted; the upper unit should not record entirely dry conditions. Moreover, MSL isotopic data for carbonates^52^ may constrain the relative contributions of escape to space versus carbonate formation to habitability’s end^1^.

**Fig. 4:**
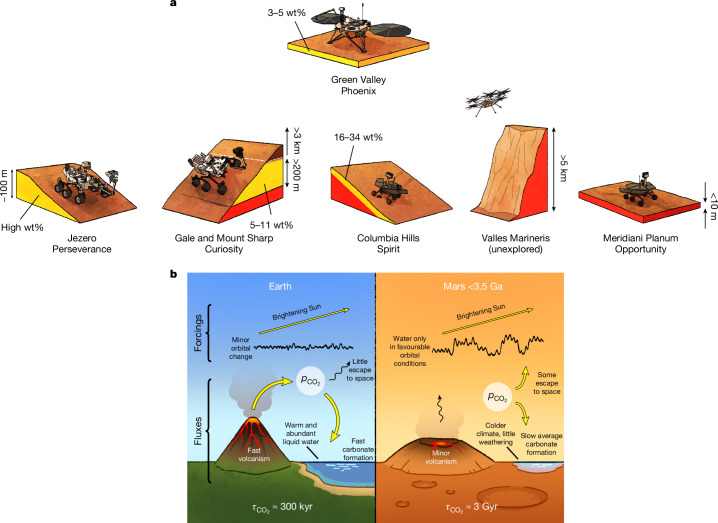
Overview of the observations and proposed model. **a**, Distribution of carbonate detections in sedimentary rocks and soil on Mars^4,18,46,55^. Yellow, abundant carbonates detected; red, no detection; brown, yet to be explored. **b**, Fluxes and feedbacks for geologic carbon and climate regulation on Mars and Earth. On Earth, temperature increase from vigorous volcanic outgassing of CO_2_ is balanced by fast carbonate formation. On Mars, slow temperature increase from solar brightening is balanced by slow (time-averaged) carbonate formation. The locally high rate of carbonate formation once liquid water is available assures that on Mars the climate has only infrequent liquid-water oases (during orbital optima) to parcel out the initial (3.5 Ga) CO_2_ endowment sparingly, and in pace with solar brightening. ${\tau }_{{{\rm{CO}}}_{2}}$, residence time in (atmosphere + surface water) reservoir.

Although MSL can disprove the scenario outlined here, it cannot prove it. In situ exploration of other mounds (for example, Valles Marineris) will ultimately be needed to determine whether the fate of Mars’s atmosphere was to be entombed in the rocks (Fig. 4a). A key test will be to determine via isotopic dating the young age of the sedimentary-rock-hosted aqueous minerals that is predicted by the model (Fig. 1), and has been inferred from orbit, but as yet has been measured for only one site on Mars (2.1 ± 0.4 Ga for aqueous jarosite at Gale using in situ K–Ar dating^53^).

In our interpretation (Fig. 4b), the post-3.5-Ga tectonic style of Mars (stagnant lid and relatively minor volcanism) acts to minimize the volcanism and weathering mechanisms that on Earth recycle carbon (as CO_2_) from sedimentary materials. Thus, post-3.5-Ga Mars tectonics does not help recycle organic and inorganic carbon-bearing condensed phases back to atmospheric CO_2_, as occurs on Earth.

Major open questions remain, including why relatively few carbonates are known^14^ from Mars’s early (pre-3.5 Ga) period of regionally integrated river networks (Methods). Perhaps sulfur dioxide (SO_2_) outgassing, rapid sedimentation or acidic fluids inhibited carbonate formation pre-3.5 Ga (refs. ^14,45,54^), or perhaps older carbonates were dissolved.

## Conclusion

Isotopic data suggest a missing carbonate sink for CO_2_ on Mars and recent discoveries by rovers suggest that this carbonate sink is now being uncovered. Key forcings for post-3.5-Ga Mars climate include solar brightening, orbital forcing and ${p}_{{{\rm{CO}}}_{2}}$ (Fig. 4). ${p}_{{{\rm{CO}}}_{2}}$ responds to volcanic outgassing, escape to space and carbonate formation. Carbonate formation and surface liquid-water availability are linked by a negative feedback that can explain fluctuating habitability on Mars (Fig. 1). This potentially explains: the time span, intermittency and patchiness of oases on Mars; the locations and total volume of the sedimentary rocks that entomb those oases (Figs. 2 and 3); the end of surface habitability on Mars; and the isotopic composition of Mars’s atmosphere. After 3.5 Ga, carbonate formation can regulate the size and duration of oases on Mars. Surface missions can test this scenario by further quantifying cryptic (undetected from orbit) carbonate.

## Methods

### Methods overview

Key drivers for Mars’s ability to sustain intermittent surface liquid water include astronomical forcing, processes that add atmospheric mass and processes that remove atmospheric mass. In turn, astronomical forcings include orbital forcing and solar brightening. Orbital forcing strongly affects the climate of Mars (high obliquity favours melting; for example, ref. ^56^), and Mars’s obliquity varies 20 times more than Earth’s over billion-year periods. The Sun brightens by about 8% Gyr^−1^. Atmospheric mass matters because very thin atmospheres preclude extensive surface liquid water on Mars, owing to a reduced greenhouse effect and more-intense evaporitic cooling^57^. Atmospheric pressure is assumed to be approximately equal to ${p}_{{{\rm{CO}}}_{2}}$ in this study. Atmospheric mass is added by volcanic outgassing, and atmospheric mass is removed by escape to space and carbonate precipitation. On the addition side, volcanism on Mars since 3.5 Ga has averaged only 10^−3^ times Earth’s current volumetric rate of volcanism (for example, ref. ^58^). Mars is less sensitive to volcanic fluctuations than Earth because Mars has more atmospheric CO_2_ mass than does Earth^59^. On the subtraction side, Mars is slowly losing CO_2_ to space^34^, and perhaps this rate was sufficiently faster in the distant past (when the Sun was much more active) to cause climate change. However, if escape to space was the sole or principal cause of atmospheric drawdown, then we would expect Mars today to either have no atmosphere or still have a fairly thick atmosphere. The observed 6 mbar (about 1% of total initially outgassed^38^), very close to water’s triple point, would require fine-tuning^5^ (Extended Data Fig. 2). In other words, escape to space does not explain why Mars’s present-day ${p}_{{{\rm{CO}}}_{2}}$ is close to the threshold for liquid water and carbonate formation. By contrast, if carbonate formation is the sole CO_2_ sink then drawdown to the threshold for liquid water (about 6 mbar) consistently occurs, suggesting a role for carbonate formation in setting ${p}_{{{\rm{CO}}}_{2}}$ (ref. ^5^; Extended Data Fig. 2). A dominant role for carbonate formation in post-3.5-Ga atmospheric loss is also suggested by isotopic data^1^. Overall, for understanding climate evolution on Mars, relative to Earth, orbital forcing matters more, escape to space matters more (but to an uncertain degree), volcanism matters less and carbonate formation matters for both worlds.

**Extended Data Fig. 3:**
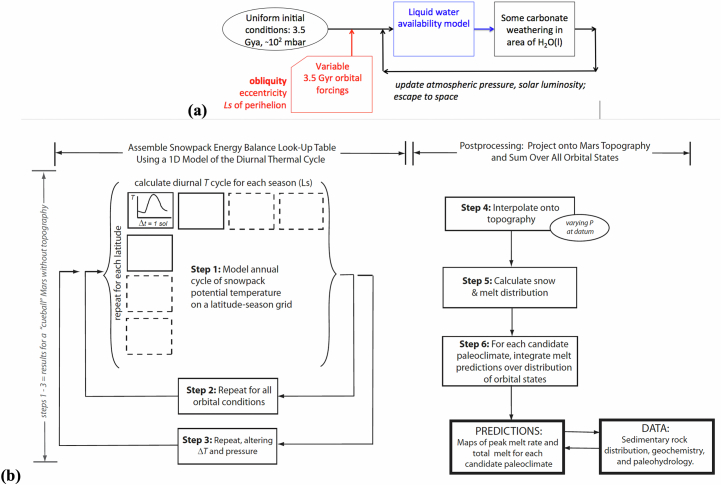
Model overview. **a**, Overall model. **b**, Liquid water availability model (modified after ref. ^36^).

We model the co-evolution of ${p}_{{{\rm{CO}}}_{2}}$ and surface and near-surface liquid-water availability (Extended Data Fig. 3). The water source in the model is snowmelt. However, the same basic model-predicted and geologically inferred spatial pattern (water at equatorial low elevations) also applies for groundwater upwelling, which requires higher temperatures^37^. The model has 1°-per-pixel spatial resolution, and covers the full Mars seasonal cycle, every 2 kyr (resolving all orbital cycles; Extended Data Fig. 7) for 3.5 Gyr. Where liquid water occurs, we allow carbonates to form at shallow (<3 m) depths within sediment, sequestering ${p}_{{{\rm{CO}}}_{2}}$ (refs. ^45,46^). Liquid water also mobilizes Mars-abundant soluble salts. Upon reprecipitation, salts cement or indurate sediment to form rock. Today Mars’s surface is dry, and water is cold-trapped at the <240-K poles. In our model, liquid water occurs at locations where (1) the annual maximum temperature is *T* > 273 K for a simulated dusty snow surface, and (2) the annual-mean snow potential sublimation rate is relatively low (a planetary cold-trap location, corresponding to where snowpack might exist). We use a parameter (*f*_snow_), corresponding to the fraction of the planet with warm-season snow or ice, to set the extent of cold traps (the definition of *f*_snow_ is discussed in detail below). The approach of assigning snow to cold traps does reasonably well when compared with precipitation-included general climate model (GCM) simulations^60^. To save computational cost, we use a surface energy balance approach. The selected surface energy balance model^36^ runs warm relative to GCM calculations^61^, so non-CO_2_ greenhouse forcing^39^ such as water-ice clouds^40^, and/or water-vapour greenhouse warming, is probably required to achieve the temperatures that the model outputs. In locations with liquid water, the rate of carbonate formation is set in our model by cation supply, which we assume is at a fixed rate (to set this rate, we use aeolian input at a fixed deposition rate). Effectively, this states that cations are added to surface water from dust deposition. This is an approximation: in reality, cation acquisition will be by water–rock interaction in the shallow subsurface. (This parameterization does not imply that a fixed percentage of each year’s aeolian input is converted in that year to carbonate; only that, without aeolian resupply, carbonate formation will eventually run out of cations). The mass percentage of the rock that is composed of carbonate is taken from MSL data.

#### Data synthesis

##### Liquid water and lithification of sediment

Mars soil is easy to cement. Mars soil contains soluble salts, which dissolve upon wetting^62,63^. Upon reprecipitation, salts indurate sediment by forming cements. Cements include sulfates, carbonates and iron oxides. For Peace-class rocks, “the cement may be essentially a [caliche composed of] sulfate”^64^. Possible liquid-water sources include snowmelt, rainfall and/or groundwater^65,66^. On Earth, sedimentary-rock accumulation is limited by sediment supply, and by tectonic subsidence to provide accommodation space^67^. By contrast, Mars post-3.5 Ga is a tectonically quiescent world where both remobilizable salts that can act as cementing agents, and aeolian sediment, are abundant^68–70^, and with abundant unfilled accommodation space (craters and canyons). On Mars (in contrast to Earth), liquid water was usually lacking and therefore was the limiting factor.

##### Location and age

We use previous maps of young sedimentary rocks^27,71–74^. Ages are estimated from crater counts^75^ (uncertain by 10^8^–10^9^ yr). Sedimentary rocks on Mars formed both post-3.5 Ga (the time period considered in this paper) and pre-3.5 Ga (for example, ref. ^76^). One hypothesis is that most of Mars’s (pre-3.5 Ga) crust is volcaniclastic^77^; consistent with this, ref. ^78^ maps a broad distribution of stratified rock. The older orbital facies^21^ appear to be carbonate poor^14–16,45^. Perhaps SO_2_ outgassing, rapid sedimentation or acidic fluids inhibited carbonate formation pre-3.5 Ga (refs. ^14,45,54,79^), or perhaps older carbonates were dissolved. These hypotheses suggest a potential role of the sulfur cycle and related pH controls of fluids, but the problem remains open.

##### Volume and thickness

Our volume and thickness analysis is very similar to that in ref. ^4^. Motivated by carbonate detections by MSL in the ‘laterally continuous sulfate’ orbital facies^21^, we estimated the thickness and volume of Mars’s laterally continuous sulfate and younger (rhythmite) facies (Extended Data Fig. 1). For each sedimentary mound, the margin was traced and an interpolated basal surface was constructed (by J. Sneed and D. P. Mayer) using triangulation-based cubic spline interpolation of Mars Orbiter Laser Altimeter^80^ 128-pixels-per-degree data. This slightly understates the volume of mounds in craters owing to the bowl shape of craters. For Meridiani Planum, we set thickness to 400 m (B. Hynek, personal communication) for areas mapped HMh, HNMe3, NMe2 and NMe1 by ref. ^72^. We neglected section 6 of Table 2 in ref. ^71^. We omit mounds at latitudes polewards of 33° (for example, Galle), which are comparatively minute. We also set aside small deposits (for example, Kaporo) that owing to their geometry are difficult to handle using triangulation-based basal-surface interpolation.

##### Duration of sedimentation

Sedimentary rock must have formed over many hundreds of million years, because many part-buried impact craters are found embedded within sediments and impacts are infrequent^81–85^. The top surface of some laterally continuous sulfate outcrops is Late Hesperian in age^72^. However, analysis of erosion rate and age suggests sedimentary-rock formation continued as late as 0.5 Ga (ref. ^28^), consistent with young formation ages for alluvial fans^86,87^ and in situ age dating^53^.

##### Carbonates in sedimentary rocks and soils

MSL has detected abundant carbonate (5–11 wt%) in the drillholes within the orbitally mapped sulfate unit that are above the rippled member of the Amapari Marker Band^4^—Tapo Caparo, Ubajara, Sequoia, Mineral King and Mammoth Lakes have been released to the Planetary Data System. These drillholes span a topographic range of about 180 m. At Meridiani, the Opportunity rover did not find abundant carbonates (its elemental analyser was insensitive to carbon). At Gusev, the Spirit rover found pre-3.5-Ga (lacustrine?) carbonate^55^. At the Phoenix landing site (Green Valley), 3–5% carbonates were detected in soil^46^: those authors inferred formation “by the interaction of atmospheric carbon dioxide with liquid water films on particle surfaces”. Carbonates can form in either warm or cold climates^88^.

##### Volcanic outgassing assessed as minor

Post-3.5-Ga volcanism is estimated at 5 × 10^7^ km^3^ (summing the partly overlapping estimates of ref. ^89^ and the ‘medium’ load of ref. ^90^, which gives an overestimate as these have some geographic overlap). Assuming a lava density of 3,000 kg m^−3^ and a magma CO_2_ mass fraction of 10^−4^ (all degassed)^58^ gives CO_2_ outgassing of (1.9 × 10^7^ km^3^ + 3.3 × 10^7^ km^3^)/1.44 × 10^8^  km^2^ × (1,000 m km^−1^) × 3,000 (kg m^−3^) × (100 ppmw) ≈ 100 kg m^−2^ ≈ 4 mbar, that is, minor (Fig. 1).

#### Details of spatially resolved long-term climate evolution model

The feedback proposed here works for any model where more water is available when Mars is warmer, including rainfall or groundwater models. We focus on a snowmelt surface liquid-water availability model because it is available.

Models of liquid-water availability including three-dimensional global climate models (GCMs) predict that (1) at >40° obliquity, water snow can pile up near the equator^60,91,92^, including at Gale Crater^42,93^, where it is more likely to melt, and (2) if groundwater upwelling occurred on Mars, then Gale is a favoured site for that upwelling^37^. Previous models for post-3.5-Ga sedimentary-rock distribution include volcanic airfall^94^, groundwater upwelling^37^ and seasonal melting^36^. As noted in the main text, groundwater upwelling can match the data if upwelling is restricted to low-latitude taliks by impermeable permafrost elsewhere^39^. Rainfall is a poor match to post-3.5-Ga geographic distributions of water-worn landforms^60,95^. Seasonal melting models offer a good fit to the observed sedimentary-rock distribution, provided that Mars was right at the cusp of habitability^36^. However, previous work gave no explanation for why Mars would be at right at the cusp of habitability, an issue that is addressed in this current work.

##### Detailed description of method

The model loop (Extended Data Fig. 3) involves building a look-up table and interpolation within the table.

Orbital forcing (red box in Extended Data Fig. 3) is imposed using the *N*-body integrator mercury6^96^. We forward-integrate the Solar System, so that the output is a statistical sampling of possible forcings experienced by past Mars, rather than a simulation of exact trajectories that past Mars could have experienced. Initial positions are taken from the modern Solar System, with a small random offset causing chaotic divergence in ≲100 Myr. Runs are postprocessed with the script of ref. ^97^ to assign solar longitude of perihelion *L*_p_ (controlled by precession angle and longitude of perihelion) and to assign obliquity. Many random initial obliquities are assigned for each *N*-body run. The result is a menu of hundreds of obliquity tracks. We do not consider tracks that end on million-year-mean obliquity >35°, as this is inconsistent with actual Mars. Future work might incorporate geologic constraints on past obliquity^98^. We randomly select tracks to drive climate evolution, sampling at 2-kyr spacing.

The climate evolution model (black box in Extended Data Fig. 3a) is initialized with a starting ${p}_{{{\rm{CO}}}_{2}}$ of 60 mbar. This is the value estimated by ref. ^99^ for “when heavy bombardment and impact erosion ended”. Carbonate sequestration is computed with inputs from a liquid-water availability model (blue box in Extended Data Fig. 3). Those inputs are (for all points on Mars) (1) the annual maximum temperature for a dusty snowpack (*T*_max_, K) and (2) a cold-trap index *f* (%). This percentage is ordinated by the annually averaged potential sublimation rate (that is, the sublimation that a snowpack would experience, if it existed). Although Mars has lost water over time^100^, we follow previous work and assume that the past snowpack was thicker but (for a given orbital forcing) not more extensive.

**Extended Data Fig. 4:**
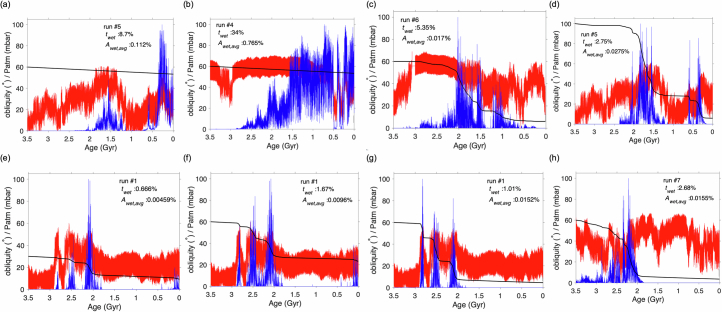
Sensitivity tests. Red line: obliquity. Black line: pCO_2_. Blue line (1 Myr average) % of the maximum area of (seasonal) surface liquid water availability (which is ~5% of planet area). *t*_*wet*_: % of time with any liquid water. *A*_*wet,avg*_: mean surface liquid water cover. **a**,**b**, No carbonate formation. **c**, Change of accumulation rate to 10× nominal value. **d**, Larger *p*_*init*_. **e**, Smaller *p*_*init*._ 30 mbar. **f**, Smaller *f*_*snow*_ = 2%. **g**, Larger *f*_*snow*_ = 20%. **h**, A different orbital forcing (otherwise as Fig. 1).

**Extended Data Fig. 5:**
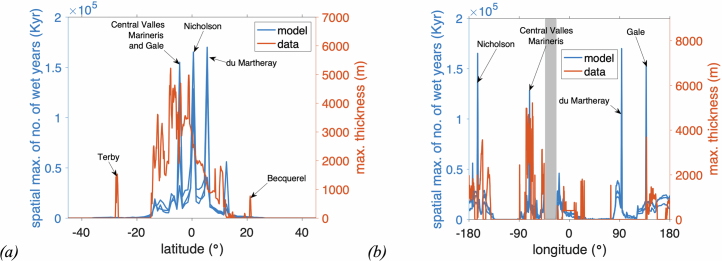
Comparison of model-predicted maximum number of wet years, and maximum sediment thickness. **a**, Variation with latitude; **b**, variation with longitude. Averages are compared in Fig. 2. Spikes at Gale, Nicholson, and Central Valles Marineris are seen, as is the absence of thick sedimentary rocks around Tharsis and Eastern Arabia. However, the model greatly underpredicts relative mound thickness around 28°S (Northern Hellas). The grey zone is masked out owing to Middle Amazonian catastrophic outburst erosion^118^. The model robustly predicts that Gale is among the maxima for sedimentary rock accumulation, as observed.

**Extended Data Fig. 6:**
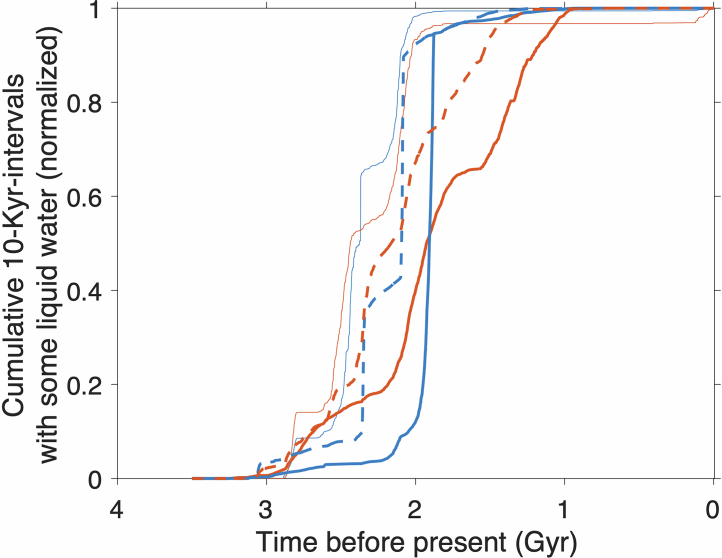
Gale is a good place to study Mars palaeoclimate, according to the model. Blue curve shows predicted Gale liquid water availability; red curve shows predicted global liquid water availability. The thin, thick, and dashed lines correspond to orbital histories 1-3 from Fig. 1. Gale captures all of the main liquid water episodes.

**Extended Data Fig. 7:**
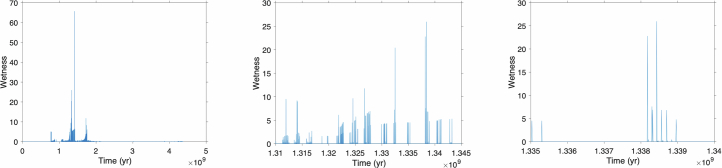
A zoom-in of the time-series of a single run for predicted liquid water at Gale. The run shown is run #1 from Fig. 1. Discretization is at 8 kyr intervals.

**Extended Data Fig. 8:**
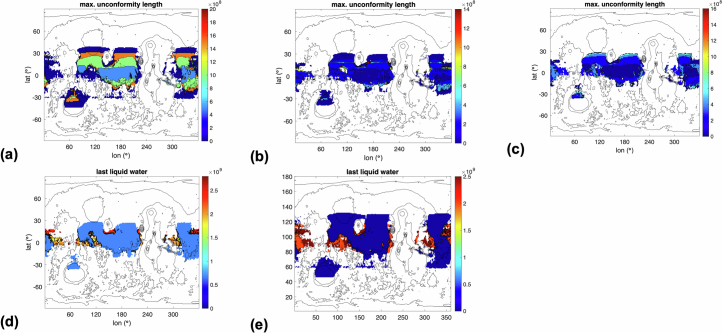
Geographic distribution of model-predicted temporal measurables. **a-c**, Maps of maximum unconformity duration (yr) for 3 different model runs (runs 1-3 from Fig. 1). The model predicts latitudinal banding in this parameter. **d**,**e**, Maps of the timing of the last surface/near-surface liquid water (yr) from two different model runs (runs 1 and 3 from Fig. 1). The model predicts that, over time, liquid water retreats from higher ground.

*T*_max_ and *f* are calculated with a surface energy balance model^36^ (summarized in ‘Snowpack surface energy balance model’ below). The surface energy balance model outputs a six-dimensional look-up-table: latitude and longitude (both 1° per pixel), pressure (approximately ${p}_{{{\rm{CO}}}_{2}}$ {24, 49, 98, 146, 293} mbar), obliquity {0, 10, 20, …, 80}°, eccentricity {0, 0.03, 0.06, 0.09, 0.115, 0.13, 0.145, 0.16} and *L*_p_, {0°, 15°, 30°, …}. Solar luminosity is fixed to 77% of modern. Temperature change owing to solar brightening is approximated using *T*^4^ scaling, that is, Δ*T* = −210 + 210 (1 + 0.078*t*)^1/4^ where 0.078 Gyr^−1^ is fractional brightening rate (fit between 3.5 Ga and 0 Ga to the results of ref. ^101^), and *t* is time in Gyr after 3.5 Ga. Liquid water is available during the warm season at a location if the (potential) snowpack temperature is >273 K and if the location is in the *f*_snow_% of Mars’s surface area where dusty snow is most stable. A threshold value of *f* < *f*_snow_ (where *f*_snow_ = 5%) is assumed, meaning that only the 5% of Mars’s area with the lowest annually averaged potential sublimation rate (the ‘cold traps’) is assigned warm-season snow and ice. Sensitivity tests for this parameter are shown in Extended Data Fig. 4f,g. A fixed freezing-point depression (1 K) is assumed, corresponding to the effect of magnesium-sulfate salts.

From these results, for each orbital forcing we pre-compute liquid-water availability for constant pressure. To do this, we snap to the closest orbital forcing in obliquity–eccentricity–*L*_p_ coordinates for each time step, including solar brightening. We do this for each of 6 atmospheric pressures (6, 24, 49, 98, 146, 293 mbar): no liquid water is permitted at 6 mbar. Linear interpolation is used for intermediate pressures. Finally, we model atmospheric pressure over time, with carbonate formation and/or escape to space. The carbonate formation rate is assumed to be limited by the cation supply (for example, ref. ^102^). This cation-limited assumption can be justified as follows. Suppose a seasonal snow supply of 2 cm yr^−1^ meltwater-equivalent, which is plausible^42^, and 25 mbar ${p}_{{{\rm{CO}}}_{2}}$. Then the solubility of CO_2_ is about 3 g kg^−1^, giving 6 g yr^−1^ of CO_2_, not including HCO_3_^−^/CO_3_^2−^. This crude calculation allows up to about 16 g m^−^^2^ yr^−1^ of FeCO_3_. In 10 Myr, this is 160 tons per m^2^ of FeCO_3_, or a drawdown of 2 bar. Thus, cations and carbonate formation kinetics, and not CO_2_ solubility or FeCO_3_ thermodynamic stability, are what limit carbonate formation^43,44^ for a sedimentation rate <0.1 cm yr^−1^ (refs. ^45,46^). Moreover, the corresponding total meltwater production is 200 km in 10 Myr, ample to explain observed water content and inferred water/rock ratios. This is just an example: similar calculations can be done for groundwater models^37^. The sediment supply rate is held constant at 30 μm yr^−1^ dense-rock-equivalent, motivated by thicknesses of rhythmic strata^29^. Sedimentary-rock density is set to 2,300 kg m^−3^ (ref. ^23^). The drawdown capability implied by 10 wt% conversion of 0.03 mm yr^−1^ of sediment into carbonate, assuming that about 30 mbar of ${p}_{{{\rm{CO}}}_{2}}$ is available for one-pass drawdown over 3.5 Gyr, implies that post-3.5-Ga Mars can only be wet for ((3,000 Pa/3.7 m s^−2^)/(0.003 m × 2,300 kg m^−^^3^ × 10 wt% × 0.3))/3.5 × 10^9^ yr = 10^−4^ of the space–time coordinates. In principle, this might correspond to small oases and long episodes, or large oases with short episodes. We do not include regional variations of cation supply rate or rock density in this study, although we acknowledge that both may have occurred.

We set the mass fraction of the daughter rock that is carbonate to 10 wt%, motivated by MSL measurements^4^. We assume that FeCO_3_ dominates^4,10^. For FeCO_3_, CO_2_ is 38% of the mass. Thus each cubic metre of sedimentary rock sequesters (2,300 kg m^−3^ × 10% × 38%) = 87 kg of CO_2_-equivalent carbon. The CO_2_ sequestration rate if the entire planet was wet would be 1 × 10^−4^ mbar yr^−1^, within a factor of 2 of Earth’s present-day rate. In our model, the planet is never wholly wet and planet-averaged carbon sequestration is always slower than on Earth. However, this slow-by-Earth-standards planet-averaged rate is still faster than other Mars sources and sinks (Fig. 4).

The climate evolution model includes atmospheric escape to space. Although escape of H and O to space from modern Mars is vigorous, almost all of these volatiles are sourced from water ice. By contrast, carbon escape from modern Mars (even including cold ion outflow) appears to be slow: about 1 mbar Gyr^−1^ (refs. ^34,35^). Carbon escape from Mars was plausibly faster when the Sun was young and more active. Current best estimates for carbon escape to space over the past 3.5 Ga are that it is slow, perhaps enough to balance post-3.5-Ga volcanic outgassing but not enough to account for the cessation of surface habitability on Mars. Escape rate is obtained from ref. ^34^ for ion escape, and adding a generous addition 20% for photochemical escape^35^. These best current estimates from data, and the most sophisticated models currently available^103^, support the minor time-integrated carbon escape reported here. However, extrapolation to solar activity levels greater than the spacecraft-era solar maximum is fraught with difficulty, not all loss channels have been measured and the activity level of the young Sun is only indirectly constrained (from data for young solar-analogue stars). Extended Data Fig. 4 shows the results with no carbon escape and the results with no carbonate formation. Ultimately more geologic (and isotopic) data will be needed to determine the fate of Mars’s atmosphere. As pointed out by ref. ^1^, a simple test remains the composition of sedimentary-rock carbonate, which MSL can measure^52^.

Other possible carbon sinks are either small or are expected to fully outgas at the relatively high temperatures relevant for surface liquid water. Polar CO_2_ ice sequesters an additional approximately 7 mbar at low obliquity, based on radar mapping of present-day polar CO_2_ ice^32^, but should outgas at higher temperature. The size of the adsorbed CO_2_ reservoir is poorly constrained (<40–100 mbar (refs. ^104,105^)), but should also passively outgas upon planet warming. There is no evidence for large deposits of CO_2_ clathrate on Mars, although they are theoretically stable. Organic matter might sequester CO_2_ via photolysis followed by incorporation of CO into organic matter via (for example) formaldehyde^106,107^. However, organic matter is present in sedimentary rocks at a concentration of about 0.5 kg m^−3^ (ref. ^108^), corresponding to only 0.1 mbar CO_2_-equivalent assuming a C/H ratio of 1:1 in the organic matter and 2 × 10^6^ km^3^ of sedimentary rocks.

In coupled (carbonate-forming) runs, melting occurs only when obliquity is ≥40°. Moreover, higher-than-average eccentricity is required for melting, and when melting occurs longitude of perihelion is usually close to equinox.

We neglect volcanic outgassing, which is probably less important than uncertainty in carbon escape to space^38^ (‘Data synthesis’). Volcanism offsets escape to space, so including volcanism would make carbonate sequestration proportionately more important to net flux. Volcanic CO_2_-outgassing fluctuations (analogous to Earth’s large igneous provinces) are unlikely to matter for Mars’s CO_2_ greenhouse effect^3^. This is due to Mars’s much larger ${p}_{{{\rm{CO}}}_{2}}$ relative to Earth, and the log-linear dependence of greenhouse warming on ${p}_{{{\rm{CO}}}_{2}}$ (ref. ^59^).

To find spatial patterns, results are resampled at 8-kyr intervals and mapped back onto latitude and longitude. We rescale to ensure that carbon is conserved. Spatial patterns shift above 24 mbar, but there is no change in spatial pattern of melt below 24 mbar, as we assume a weak greenhouse effect below 24 mbar. Perennial CO_2_ ice is neglected, which is acceptable because for habitable conditions (high obliquity) there is no perennial CO_2_.

We neglect late-stage true polar wander, which was probably small^39,109^.

Model output depends on parameters, including *f*_snow_, accumulation rate, and the initial partial pressure of CO_2_ (*p*_init_). Raising *p*_init_ causes more carbonate to form, but (owing to the negative feedback) makes little difference to the habitability timescales.

Simple models have limitations that point the way for future work. Although assigning snow to ‘cold traps’ reproduces three-dimensional model output quite well^60^, it is not true in detail. Our model neglects horizontal heat transport by the atmosphere: this is acceptable at present-day pressure, but less so for higher ${p}_{{{\rm{CO}}}_{2}}$.

##### Sensitivity tests

Runs with escape to space, but without carbonate formation, can (owing to orbital change) give intermittent surface liquid water (Extended Data Fig. 4). Therefore, the inference that surface liquid water was intermittent does not by itself require carbonate formation. However, for these no-carbonate-formation runs to match modern ${p}_{{{\rm{CO}}}_{2}}$ challenges isotopic constraints and requires fine-tuning of the initial ${p}_{{{\rm{CO}}}_{2}}$ and escape rate (Extended Data Fig. 2), motivating a search for an explanation that does not require fine-tuning.

#### Snowpack surface energy balance model

Previous work on seasonal melting of dusty Mars snowpack includes refs. ^48,110,111^. We use the model of ref. ^36^. A uniform dusty snowpack albedo of 0.28 is assumed^36,112^. Thermal inertia is set to 250 J m^2^ K^−1^ s^−1/2^. The background-atmosphere relative humidity is 0.25. Look-up tables for greenhouse forcing and Rayleigh scattering are from a line-by-line code^113^, using a prescribed atmospheric profile starting at the surface *T* of interest (dry adiabatic troposphere patched to an isothermal stratosphere at 167 K). The temperature at the base of the atmospheric column is set to the diurnal-average surface temperature. Forced and free sensible heat transfer and sublimation loss equations follow those of ref. ^114^. (Reference ^112^ modifies these rates at the tens of per cent level). These equations require an atmospheric temperature fitting parameter, *b*_DB_, which is obtained as a function of pressure using GCM runs at 7 mbar, 50 mbar and 80 mbar (model of refs. ^115,116^). GCM runs are also used to parameterize the relationship between wind speed and atmospheric pressure. Temperatures are calculated as a function of depth within the dusty snowpack. The solid-state greenhouse effect is included, but is not important. Neither drainage of melt nor advection of heat corresponding to the ablation of the snowpack are included. The surface topographic slope is zero (see ref. ^48^. for steep-slope calculations). For each of 24 ‘seasons’ equally spaced in solar longitude *L*_s_, the model is run for several sols until temperature has converged to <0.01 K and daily melt production has converged, or until 8 sols have elapsed, whichever is sooner. The 8-sol cut-off is needed because polar summer melting can proceed indefinitely. Mars’s orbit is eccentric and Kepler’s equation is used to relate *L*_s_ to time elapsed.

## Online content

Any methods, additional references, Nature Portfolio reporting summaries, source data, extended data, supplementary information, acknowledgements, peer review information; details of author contributions and competing interests; and statements of data and code availability are available at 10.1038/s41586-025-09161-1.

## Figures and Tables

**Extended Data Table 1:**
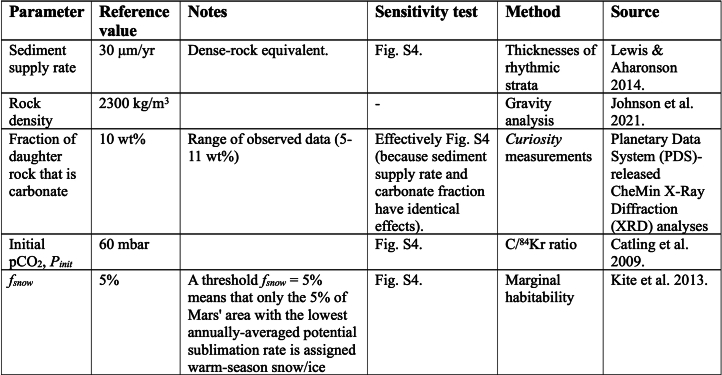
Selected model parameters

Table shows basis for fiducial values. See ref. ^36^ for details of snowmelt model parameters.

**Extended Data Table 2:**
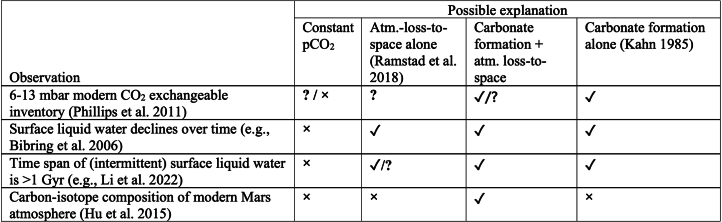
Comparison of models and data for post-3.5 Ga Mars climate evolution

Geologic and isotopic constraints compared to models of atmospheric evolution. ✓ = this model can explain this observation;? = special circumstances are required to produce this observation from this model; × = this model does not plausibly explain this observation. Chaotic orbital forcing is assumed. See Extended Data Fig. 2 for further explanation.

## Data Availability

All data are available through the NASA Planetary Data System (https://pds.nasa.gov/). The topographic contours on the maps shown in Fig. 3, and Extended Data Figs. 1 and 8 are made using MATLAB from publicly available Mars Orbiter Laser Altimeter gridded records (https://pds-geosciences.wustl.edu/missions/mgs/megdr.html).

## Code availability

Our Mars climate evolution model code, together with our analysis scripts, is open-sourced on *Zenodo* at 10.5281/zenodo.11489512 (ref. ^117^).
